# SyRACT: zero-shot biomedical document-level relation extraction with synergistic RAG and CoT

**DOI:** 10.1093/bioinformatics/btaf356

**Published:** 2025-06-19

**Authors:** Xin Dong, Di Zhao, Jiana Meng, Bocheng Guo, Hongfei Lin

**Affiliations:** School of Computer Science and Engineering, Dalian Minzu University, Liaoning 116600, China; School of Computer Science and Engineering, Dalian Minzu University, Liaoning 116600, China; School of Computer Science and Technology, Dalian University of Technology, Liaoning 116024, China; School of Computer Science and Engineering, Dalian Minzu University, Liaoning 116600, China; School of Computer Science and Engineering, Dalian Minzu University, Liaoning 116600, China; School of Computer Science and Technology, Dalian University of Technology, Liaoning 116024, China

## Abstract

**Motivation:**

With the advancement of large language models (LLMs), the field of biomedical document-level relation extraction (BioDocRE) has encountered new opportunities. However, LLMs often face challenges such as hallucinated generation, insufficient reasoning capabilities, and a lack of interpretability when performing relation extraction tasks.

**Results:**

To address these issues, we propose the SyRACT (Synergistic Retrieval Augmented Generation and Chain of Thought) framework for high precision relation extraction in biomedical documents. This framework is built around three core strategies: (i) reframing the relation extraction task as a question answering problem to better align with the processing logic of LLMs; (ii) leveraging an external database constructed from PubMed to provide LLMs with rich and reliable contextual information, thus mitigating hallucination generation; and (iii) construct a specific Chain of Thought for BioDocRE tasks, thereby enhancing the model’s reasoning ability and the interpretability of its output. We validated this approach on three biomedical relation extraction datasets: CDR, GDA, and ADE. Experimental results show that the SyRACT model improves *F*1 scores by 11.04%, 9.10%, and 41.00% on three datasets, respectively, compared to the DocRE method, which uses standard prompts for LLMs.

**Availability and implementation:**

Our source code and data are available at https://github.com/donggggxin/SyRACT.

## 1 Introduction

**Figure 1.**
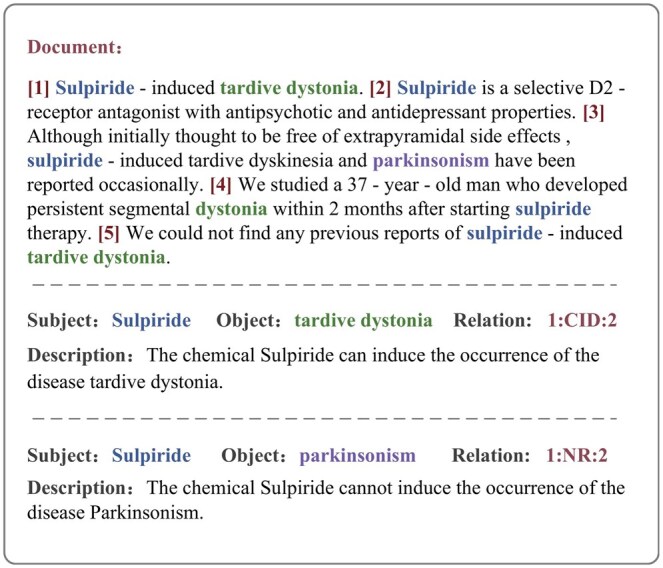
An example from the CDR dataset. The label “1:CID:2” denotes Chemical-Induced Disease, while “1:NR:2” indicates No Relation.

Biomedical relation extraction, as one of the key foundational tasks in biomedical information mining, can accurately identify and extract the relation between entities from these complex textual data, such as the relation between chemicals and diseases, genes and diseases, drugs and adverse reactions, etc. These relations are not limited to causal relation (e.g. “cause”), but also include associative relation (e.g. “related to”). Accurate identification of these relation is crucial for the systematic organization and discovery of biomedical knowledge. Figure 1 shows an example from the CDR dataset.

Existing methods for relation extraction can be broadly divided into two categories: sentence-level (Chen *et al.* 2022, Xu *et al.* 2024) and document-level relation extraction (DocRE) (Huang *et al.* 2022, Ma *et al.* 2023b). Earlier research predominantly focused on sentence-level relation extraction. In real world contexts, however, relation between entities are frequently spread across several sentences. As a result, since 2019, DocRE has gained significant attention and emerged as a research hotspot.

In recent years, DocRE methods can be mainly categorized into sequence-based approaches (Zheng *et al.* 2018) and graph-based approaches (Zhou *et al.* 2020). However, most of these methods rely on the model to automatically learn the representation of relation during training, without explicitly guiding the model to focus on important contextual information or entity types. As a result, the model fails to explicitly attend to crucial signals for the extraction task at the appropriate moments, leading to vague or inaccurate inferences. To address these challenges, the SAIS method has emerged (Xiao *et al.* 2022), which enhances the model’s ability to capture context and entity types through explicit supervision, thereby improving the quality and interpretability of relation extraction.

Meanwhile, large language models (LLMs) are evolving with remarkable capabilities, reigniting interest in the potential of DocRE through their strong reasoning abilities. In response to this, the AutoRE (Xue *et al.* 2024) model was developed, introducing a novel end-to-end architecture and a new paradigm for relation extraction. This paradigm overcomes the limitations of existing methods that rely on predefined relation choices, but due to the limitations of training data coverage, its ability to generalize when handling unknown relations remains insufficient.

In addition, LLMs face numerous challenges in other practical applications, particularly issues such as hallucination (Ni *et al.* 2024), insufficient reasoning abilities (Trivedi *et al.* 2023), and lack of interpretability (Wang *et al.* 2020), which to some extent limit their performance in DocRE tasks. Notably, existing research indicates that retrieval augmented generation (RAG) techniques can dynamically inject reliable external context into LLMs, effectively mitigating the hallucination problem (Fan *et al.* 2024). At the same time, chain of thought (CoT) methods have also been shown to help models perform step by step reasoning, enhancing their ability to solve complex logical problems (Lee *et al.* 2024).

Although researchers have made certain progress with the methods mentioned above, existing LLM-based biomedical document-level relation extraction (BioDocRE) methods still face some limitations. First, a single technique struggles to address hallucination issues, improves reasoning abilities, and enhances model interpretability within the same framework. Second, while LLMs have shown strong potential in natural language processing tasks, fine tuning them for vertical domains such as biomedical fields still faces challenges related to high computational costs and the need for high quality data. Therefore, existing BioDocRE methods lack a unified framework that can simultaneously address hallucination problems, enhance reasoning abilities, and improve model interpretability while saving resources.

Based on these reasons, in this article, we propose the SyRACT (Synergistic Retrieval Augmented Generation and Chain of Thought) framework, which synergistically applies RAG and CoT techniques to zero-shot BioDocRE tasks. The key contributions of this framework are highlighted in the following three aspects.

- Reframe the DocRE task as a question answering problem, aligning it more closely with the reasoning mechanisms of LLMs, and fully leveraging their natural language understanding capabilities.
- Apply RAG techniques to dynamically retrieve relevant information before generating answers, providing reliable external context to mitigate the risk of hallucinations in LLMs, while enhancing their performance in vertical domains like biomedicine.
- Utilize CoT techniques to strengthen the step by step reasoning abilities of LLMs, further improving model accuracy and the interpretability of results, allowing for more effective handling of complex logical reasoning tasks.

## 2 Related work

### 2.1 Document-level relation extraction

DocRE aims to identify and extract complex relation between entities in long texts, a task that is crucial for downstream tasks such as knowledge graph construction. As research has progressed, many scholars have continuously explored new methods to improve the accuracy and robustness of DocRE. Zhao *et al.* (2023) proposed a model that combines the T5 model with prompt learning. Through prompt learning, this approach narrows the task gap between the pre-training and fine tuning stages of pre-trained language models and simplifies the complex workflow of traditional DocRE.

With the rapid development of LLMs, their advantage of large scale pre-training has attracted an increasing number of researchers to shift their focus toward exploring the potential of LLMs in DocRE tasks. Li *et al.* (2023b) introduced a semi-automatic data augmentation method that combines LLMs and a Natural Language Inference module to address the challenges of manual annotation and the difficulty of capturing long-tail relation types in traditional DocRE. Additionally, to reduce reliance on fully annotated data, ZeroDocRTE introduces a zero-shot DocRE framework (Sun *et al.* 2024). This framework guides the model to generate annotated data through chain based retrieval prompts, and uses the denoised synthetic data to fine tune the LLMs, enabling efficient extraction of document-level relation triplets.

At the same time, some researchers (Jiang *et al.* 2024) have attempted to use LLMs for generative relation extraction (GRE). This method allows LLMs to directly perform zero-shot relation extraction by understanding textual information, without the need for additional training. However, traditional LLMs often struggle to accurately recognize all relevant medical concepts when processing domain-specific terms in the biomedical field. In response to this, Bhattarai *et al.* (2024) proposed mapping Unified Medical Language System (UMLS) concepts to clinical texts and dynamically generating prompts based on these concepts to guide GPT models in entity and relation extraction.

Our research similarly leverages LLMs for zero-shot relation extraction to address the unique requirements of biomedical texts.

### 2.2 Retrieval augmented generation

RAG was first introduced by Lewis *et al.* (2020), significantly alleviates hallucinations in LLMs caused by outdated knowledge or insufficient domain-specific information. In recent years, RAG has shown broad application potential in various natural language processing tasks. Xie *et al.* (2025) proposed the Retrieval Augmented Instruction Tuning (RA-IT) framework, which incorporates retrieval augmented mechanisms into instruction tuning by dynamically retrieving semantically similar contextual examples for each training sample, significantly improving performance on open-domain named entity recognition tasks. Kresevic *et al.* (2024) applied RAG to the interpretation of medical clinical guidelines, using semantic retrieval and matching between complex medical questions and guideline content, effectively enhancing the model’s accuracy in professional medical scenarios.

Inspired by these studies, this article leverages the biomedical knowledge base PubMed to retrieve relevant documents as an external knowledge source, further enhancing the performance of the model in BioDocRE tasks.

### 2.3 Chain of thought

As tasks become more complex, traditional standard prompts are insufficient to handle intricate logical reasoning tasks. To address this issue, Wei *et al.* (2022) proposed Chain of Thought Prompting, a method that introduces a series of intermediate reasoning steps during the prompting process, helping the model break down complex problems. Unlike Wei *et al.* (2022) and Ma *et al.* (2023a) who focused on few-shot CoT, Kojima *et al.* (2022) explored its application in zero-shot scenarios, significantly improving zero-shot LLMs performance by adding the simple prompt, “Let’s think step by step”. To further optimize CoT, Zhang *et al.* (2023) introduced Auto CoT, which enhances LLMs reasoning ability through question clustering and automatic generation of reasoning chains, eliminating the need for manual prompts. Although CoT has achieved remarkable success across various NLP tasks, its application in BioDocRE remains to be further validated.

## 3 Materials and methods

**Figure 2.**
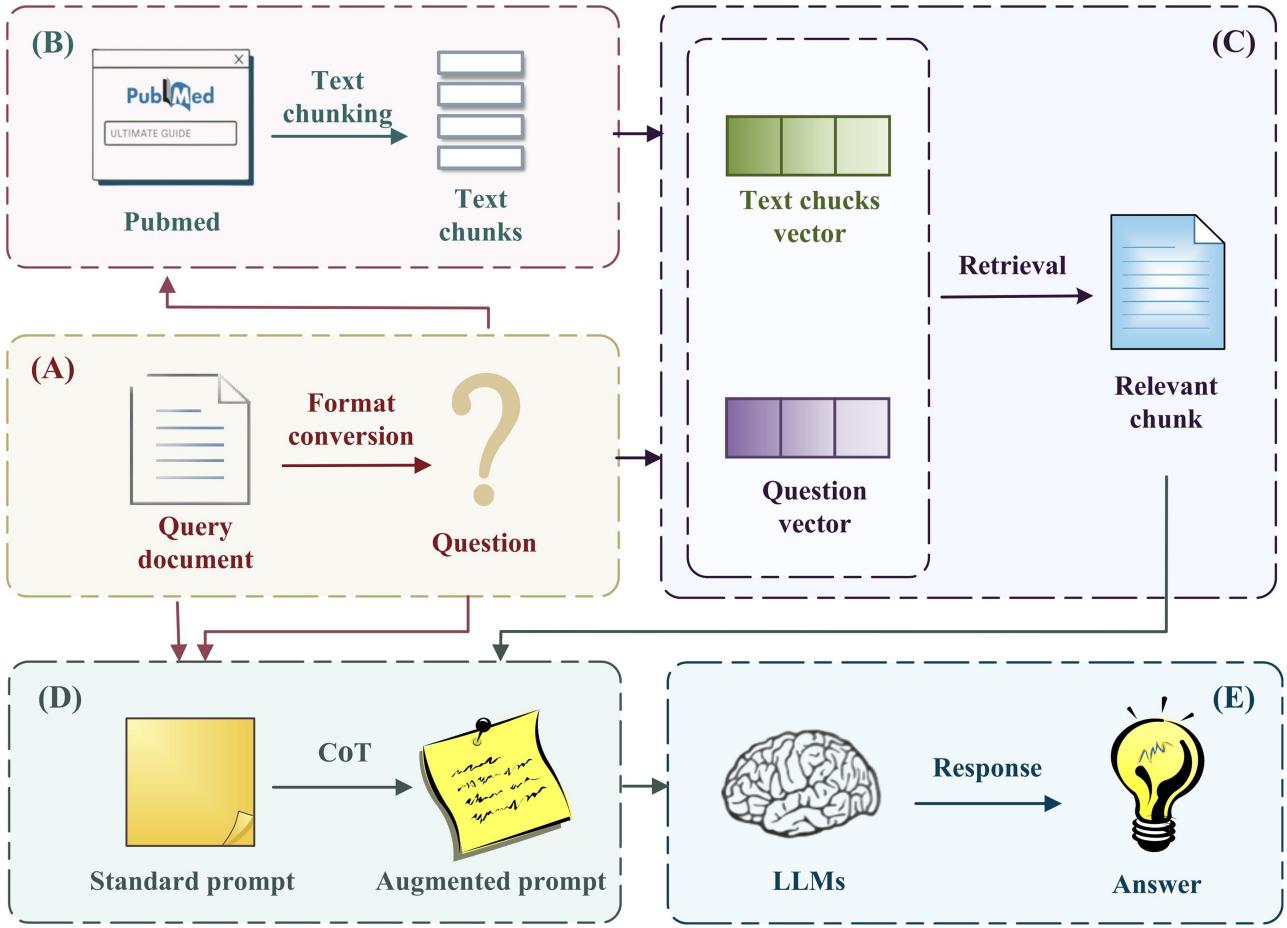
The modeling architecture of our proposed methods.

The SyRACT framework mainly consists of five modules, namely Task Reconstruction, Data Collection and Preprocessing, Retrieval, CoT Instruction Enhancement, and Generation. The overall architecture is shown in Fig. 2.

### 3.1 Task reconstruction

Traditional BioDocRE methods typically frame the task as a classification problem, aiming to identify different types of relations in a given biomedical document. However, with the rapid development of LLMs, researchers have begun to explore the potential of these models in the domain of DocRE. Through in-depth analysis of BioDocRE datasets, we found that most existing datasets in this field are binary classification problems targeting specific domains (as shown in Table 1). Specifically, the tasks in these datasets can be simplified to determining whether a specific relation exists between entities (e.g. chemical-induced disease occurrence, gene disease association, drug induced adverse reactions, etc.), without the need to consider complex multi-class classification.

**Table 1.** Display of relation types for each dataset.

Dataset	Relation	Relation description
CDR	1:CID:2	Chemical induces disease occurrence
	1:NR:2	Chemical does not induce disease occurrence
GDA	1:GDA:2	Gene is related to disease
	1:NR:2	Gene is not related to disease
ADE	Drug-ADE	Drug-induced adverse drug reactions

**Figure 3.**
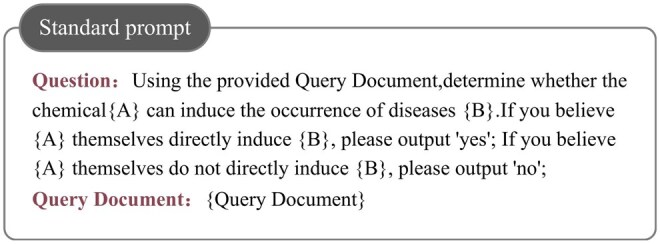
Example of standard prompt.

Based on this insight, this article proposes an innovative relation extraction method that redefines the traditional relation extraction task within a question answering framework. Specifically, we construct targeted questions *Q* based on entities $e_{1}$ and $e_{2}$ and their category information, such as “Does chemical $e_{1}$ induce disease $e_{2}$?” to guide the LLM in making relation judgments. This approach aligns more closely with the design principles and working logic of LLMs, helping to improve the accuracy and efficiency of relation extraction. Meanwhile, the question *Q* is combined with the query document to form the standard prompt used in this study, as illustrated in Fig. 3.

### 3.2 Data collection and preprocessing

PubMed is a comprehensive database of biomedical literature, with articles that are highly reliable and authoritative. Furthermore, PubMed provides the MeSH (Medical Subject Headings) terminology system, which effectively standardizes and classifies entities, enabling more precise retrieval and data integration. This addresses the issue of multiple mentions of the same entity in DocRE. Therefore, we utilize the PubMed platform to construct an external database.

Specifically, for the entities $e_{1}$ and $e_{2}$ involved in question *Q*, we first retrieved the corresponding MeSH terms $\mathrm{mes}h_{1}$ and $\mathrm{mes}h_{2}$ from the MeSH database. For entities without corresponding MeSH terms in the database (such as specific genes), we used their official symbols as the MeSH terms. Subsequently, we queried the PubMed database for unique identifiers (PMIDs) of articles where both terms $\mathrm{mes}h_{1}$ and $\mathrm{mes}h_{2}$ appear together in the title or abstract, resulting in $\mathrm{PMIDs}={\left\{\mathrm{PMI}D_{i}\right\}}_{i=1}^{P}$, where P represents the number of articles. To avoid redundancy in the external knowledge base, if P>10, we select only the top 10 PMIDs based on the Best Match ranking. If no article contains both entities simultaneously, we query each entity separately, retrieve the top five PMIDs based on the Best Match ranking for each, and then merge the final results.

After obtaining the PMID, we further extracted the corresponding abstract information. For each abstract $Abs_{i}={\left\{abs_{i}\right\}}_{i=1}^{M}$, where *M* represents the number of paragraphs in the abstract, we first employed a paragraph-based segmentation method to improve the accuracy of text processing. To further optimize the identification of similar text chunks, we additionally applied two different segmentation strategies: sentence-level and sliding window-level segmentation. Sentence-level segmentation involved splitting each abstract paragraph $abs_{i}={\left\{s_{i}\right\}}_{i=1}^{N}$ into individual sentences $s_{1},s_{2},\ldots ,s_{n}$. The sliding window segmentation method groups *k* consecutive sentences together, and in this study, we set k=3 with a sliding step size of d=2, resulting in a one sentence overlap between windows. For example, for the sentence group [$s_{1},s_{2},\ldots ,s_{N}$], the sliding window generates $c_{1}=[s_{1},s_{2},s_{3}],\;c_{2}=[s_{2},s_{3},s_{4}],\;c_{N-2}=[s_{N-2},s_{N-1},s_{N}]$, forming a sequence of sliding window sentence groups $C_{i}={\left\{c_{i}\right\}}_{i=1}^{N-2}$. This approach ensures that each newly generated sentence chunk contains three consecutive sentences from the original text.

Finally, we merged all text chunks generated from the three segmentation strategies and reordered and saved them according to their unique identifiers to ensure data uniqueness and traceability. This process not only improved data organization efficiency but also laid a solid foundation for the subsequent retrieval of similar.

### 3.3 Retrieval

To evaluate the similarity between question *Q* and text chunks, this study utilized the all-MiniLM-L6-v2 model as the sentence-BERT (SBERT) model to encode the question *Q* and text chunks, generating their high dimensional vector representations. Specifically, we input question *Q* and each text chunk into the SBERT model to generate the corresponding vector representations. Then, the cosine similarity between these vectors was calculated to quantify the similarity between the question and the text chunks. Through this method, we were able to identify the text chunk most semantically similar to the given question, referred to as the relevant chunk. The relevant chunk was then selected as the external knowledge for constructing the subsequent augmented prompt.

### 3.4 CoT instruction enhancement

To further enhance the reasoning capabilities of LLMs and improve the interpretability of their outputs, this study introduces the CoT method. The CoT method aims to guide LLMs in logical reasoning and deep thinking, thereby increasing the transparency of their decision making process. During the implementation process, we first set up the initial version of CoT based on the task background and settings.

For example: 1. Define the concept of chemically induced diseases clearly. 2. Determine whether chemical entity $e_{1}$ induces the occurrence of disease entity $e_{2}$.

**Figure 4.**
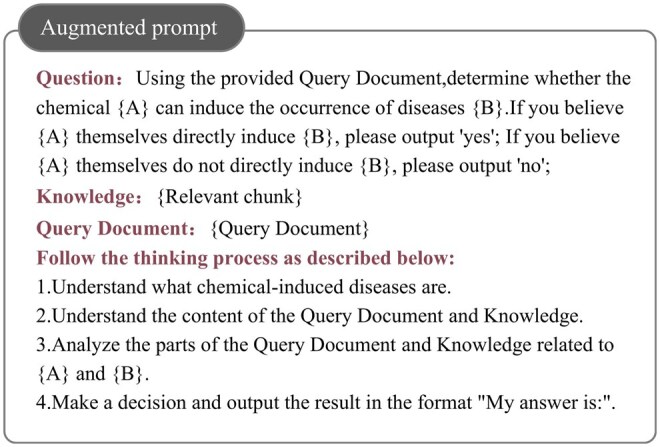
Example of augmented prompt.

In order to adapt to data bias, the subsequent adjustments and optimizations involve using only 3–5 unlabeled training samples for adjusting and optimizing the CoT, which results in the final version of CoT (Zhang *et al.* 2024). This is then combined with the standard prompt along with the relevant retrieved chunk to create the augmented prompt, as illustrated in Fig. 4.

### 3.5 Generation

We input the enhanced prompts into the LLMs and set a clear output format specification for it. Specifically, while we do not interfere with the reasoning outputs generated by the LLMs during the chain of thought process, to improve the efficiency of evaluation and analysis, we require the LLMs to follow a specified format when providing its final answer, namely prefixing the result with “My answer is:”. This standardized output format not only facilitates easier extraction, processing, and analysis of the generated results during subsequent evaluations, but also ensures consistency across outputs from different models, making it easier to compare their performance on various tasks.

## 4 Results

### 4.1 Datasets and evaluation metrics

This study validates the model using three biomedical relation extraction datasets: the Chemical Disease Reactions (CDR) dataset, the Gene Disease Associations (GDA) dataset, and the Adverse Drug Events (ADE) Corpus. Since our focus is on zero-shot scenarios, we only use the test sets for the CDR and GDA datasets, which clearly distinguish between training, validation, and test sets. For the ADE dataset, which does not provide such distinctions, we utilize the entire dataset.

The CDR is a DocRE dataset consisting of abstracts from PubMed articles, primarily focusing on binary interactions between chemicals and diseases (Li *et al.* 2016). Similarly, the GDA is a DocRE dataset that emphasizes binary relation between genes and diseases (Wu *et al.* 2019). The ADE Corpus comprises case reports, reflecting the relation between drugs and adverse reactions (Gurulingappa *et al.* 2012). The specific sizes of each dataset are shown in Table 2, and the relation descriptions are shown in Table 1. It is important to emphasize that although the ADE dataset annotates only Drug-ADE one type of relation, the prediction task requires determining whether a drug causes an adverse event. Therefore, all relations evaluated in this study are binary in nature.

**Table 2.** Details of various datasets.

Dataset	Document	Total instances
CDR	500	5405
GDA	1000	5222
ADE	4272	6821

We evaluated the performance of SyRACT using Precision (P), Recall (R), and *F*1 score (*F*1) as evaluation metrics. Additionally, to improve the stability of the results, we introduced standard deviation as a supplementary measure.

### 4.2 Baselines

We compared our method with the following models: PTR (Han *et al.* 2022), a rule-based Prompt Tuning with Rules method, combines multiple sub-prompts into task specific prompts using logical rules, introducing prior knowledge to assist model learning. In our experiments, we chose BioBERT as the pre-trained language model and used random seeds to randomly sample three subsets for 16-shot testing, reducing instability caused by random data sampling. Guo *et al.* (2024) proposed a few-shot relation extraction approach enhances model performance in few-shot scenarios by integrating synthetic data augmentation strategies and domain information. According to the article, we used BioLinkBERT-large as the pre-trained language model and similarly performed 16-shot experiments three times, taking the average. Closed GRE (Li *et al.* 2023a), by inputting the source text (sentences or documents), head and tail entities, as well as predefined relation types into LLMs for relation extraction, in this article, we directly used the experimental results on the CDR dataset provided by Jiang *et al.* (2024). Bhattarai *et al.* (2024) proposed a framework combining UMLS with GPT based generative models to improve clinical entity and relation extraction tasks.

Additionally, we conducted three standard prompt experiments on CDR and GDA using T5-XL, ChatGLM3-6B, GPT-3.5-turbo, Qwen2-1.5B-Instruct, and Mistral-7B-Instruct-v0.2. The results are shown in Table 3. Since this study aims to achieve a balanced performance between precision and recall, we adopted the *F*1 score as the primary evaluation metric. It can be observed that GPT-3.5-turbo demonstrates superior and more stable overall performance compared to the other models. Therefore, we selected GPT-3.5-turbo as the LLM for our experiments. Notably, in certain application scenarios where recall is more critical, other models with better recall performance should be considered.

**Table 3.** Result comparisons on CDR and GDA.

	CDR			GDA		
Model	*P* (%)	*R* (%)	*F*1 (%)	*P* (%)	*R* (%)	*F*1 (%)
Few-shot (16-shot)						
PTR (Han *et al.* 2022)	51.22±3.4	40.06±3.9	44.94±3.7	46.24±1.4	62.92±3.6	53.28±1.8
Guo *et al.* (2024)	51.21±1.1	53.85±3.5	52.44±1.2	44.24±0.9	68.80±4.9	53.86±2.3
Zero-shot (LLMs)						
Closed GRE (Jiang *et al.* 2024)	**58.80**	58.70	58.80	–	–	–
T5-XL	33.39±5.15	81.99±2.62	47.12±4.72	32.91±0.89	70.81±0.24	44.93±0.88
ChatGLM3-6B	38.19±18.45	58.44±28.05	35.86±3.74	35.09±4.7	75.43±15.91	46.65±1.04
GPT-3.5-turbo	38.40±1.06	**82.20 ± 8.69**	52.15±0.78	33.97±0.27	71.34±2.23	46.01±0.22
Qwen2-1.5B-instruct	28.58±2.23	67.87±6.34	39.98±1.10	31.75±1.36	**84.12 ± 7.22**	45.94±0.34
Mistral-7B-Instruct-v0.2	44.06±2.14	49.65±5.36	46.65±3.57	31.96±3.58	56.52±9.32	40.11±0.44
**SyRACT (ours)**	53.59±0.89	77.05±1.86	**63.19 ± 0.18**	**47.08 ± 1.45**	66.54±1.24	**55.11 ± 0.57**

Bold values indicate the best performance in each column.

##  

### 4.3 Results on CDR

As shown in Table 3, our method achieved remarkable performance in the zero-shot setting, outperforming the standard prompting approach of inputting the query document and question *Q* into the LLMs for relation extraction, with an *F*1 score improvement of 11.04%. Moreover, in the zero-shot setting, our method even outperformed some few-shot methods in the 16-shot scenario, demonstrating that our approach not only has strong generalization capabilities but also effectively handles complex reasoning tasks to a certain extent.

### 4.4 Results on GDA

As shown in Table 3, our method also performed exceptionally well on the GDA dataset, with an *F*1 score 9.10% higher than the standard approach of inputting the query document and question *Q* into the LLM for relation classification. Furthermore, our method surpassed the performance of some few-shot approaches in the 16-shot experiments. This result further validates that our method, by combining enhanced retrieval and reasoning mechanisms, is better at capturing complex relations and contextual information between entities, and has significantly improved generalization ability across various scenarios.

### 4.5 Results on ADE

In this dataset, we conducted an in-depth comparison with the new framework proposed in reference (Bhattarai *et al.* 2024), which enhances GPT model performance by incorporating the UMLS knowledge base. The specific results are shown in Table 4, where results marked with * are sourced from Bhattarai *et al.* (2024). The comparison demonstrates that, using the same LLM, our method achieved a significant performance improvement over the standard prompting approach, with a 41% increase. Additionally, for the two UMLS-based enhancement methods (1) directly introducing UMLS concepts into the LLM and (2) using UMLS as an external database for RAG, our model achieved a 34% and 36% performance improvement, respectively. This result highlights the superiority of our method when integrating external knowledge.

**Table 4.** Result comparisons on ADE Croups.

Model	*P*	*R*	*F1*
GPT-3.5-turbo*	0.57	0.53	0.55
GPT-4-32k*	**1.0**	0.73	0.84
GPT-3.5-turbo + UMLS*	0.60	0.65	0.62
RAG w/GPT-3.5-turbo*	0.62	0.61	0.60
RAG w/GPT-4-32k*	**1.0**	0.73	0.84
SyRACT(Ours)	**1.0**	**0.92**	**0.96**

Bolded values indicate the best performance in each column.

Results marked with * are reported by Bhattarai *et al.* (2024).

Furthermore, when using the GPT-4-32k model, our method achieved a 12% improvement over both the standard prompting approach and the method using UMLS as an external database for RAG. This increase not only demonstrates a significant improvement in prediction accuracy but also showcases the advantages of our method in resource utilization.

## 5 Discussion

### 5.1 Performance of each functional module

To verify the effectiveness of our proposed method, we conducted ablation studies on both the CDR and GDA datasets, with the results presented in Table 5.

**Table 5.** Ablation study results on CDR and GDA.

	CDR			GDA		
Model	*P* (%)	*R* (%)	*F*1 (%)	*P* (%)	*R* (%)	*F*1 (%)
SyRACT (ours)	**53.59**	77.05	**63.19**	**47.08**	66.54	**55.11**
w/o RAG	49.28	**80.21**	61.05	37.78	73.57	49.92
w/o CoT	46.72	66.85	55.00	41.62	**74.23**	53.34

Bolded values indicate the best performance in each column.

#### 5.1.1 Impact of RAG

In Table 5, “w/o RAG” indicates that our model excluded the RAG method, relying solely on the CoT technique. The results show that without RAG, the *F*1 scores of the model decreased by 2.14% and 5.19% on the CDR and GDA datasets, respectively. This change reflects the fact that the BioDocRE task involves a large amount of complex domain-specific knowledge, and relying solely on the input content for reasoning can lead to knowledge gaps, which in turn affect the model’s performance. This further highlights the crucial role of the RAG method in this framework, as it incorporates external knowledge, providing richer contextual information and effectively filling knowledge gaps, thereby improving model performance.

#### 5.1.2 Impact of CoT

In Table 5, “w/o CoT” indicates that our model did not use the CoT method, relying solely on the RAG method. We observe that the *F*1 scores on the CDR and GDA datasets decreased by 8.19% and 1.77%, respectively. This result further validates the effectiveness of the CoT technique, which guides the LLMs to gradually break down the BioDocRE tasks, enhancing the reasoning depth of LLMs in BioDocRE tasks. This allows the model to better capture hidden semantic clues and entity relation within sentences or documents, thereby improving the overall performance of the model.

### 5.2 Impact of chunking strategies

**Figure 5.**
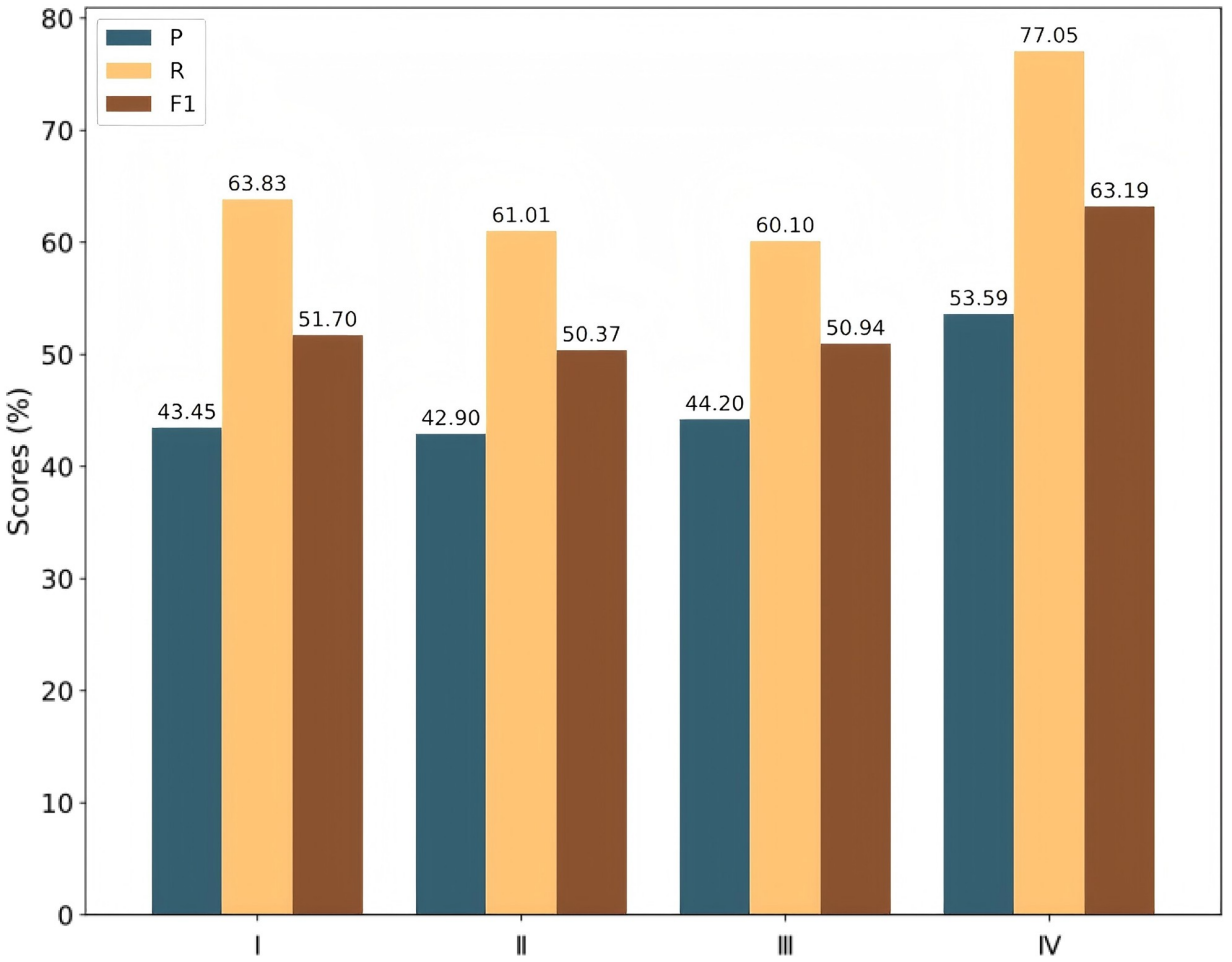
The impact of different chunking strategies.

To explore more suitable document segmentation strategies for external knowledge bases in BioDocRE tasks and optimize the model’s external knowledge retrieval and reasoning capabilities, we designed and compared four different segmentation methods: sentence-based segmentation, sliding window segmentation k=3, d=2, paragraph-based segmentation, and a hybrid of the three methods. In Fig. 5, we denote these methods as I, II, III, and IV, respectively.

As shown in Fig. 5, the *F*1 scores of methods I, II, and III are all between 50% and 52%, showing relatively close performance. However, when we applied the mixed segmentation method IV, the *F*1 score significantly increased to 63%, representing an improvement of nearly 12% compared to the other methods. This result indicates that the mixed segmentation strategy we employed is able to more effectively capture the multi-level semantic information and contextual associations within the document, thereby enhancing the model’s performance in BioDocRE tasks.

### 5.3 Impact of different prompts

**Figure 6.**
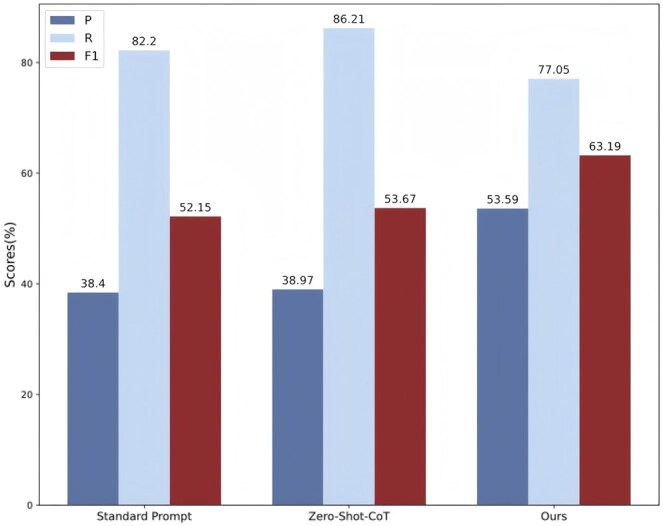
The impact of different prompts.

Additionally, to thoroughly validate the effectiveness of the CoT prompting strategy proposed for BioDocRE, we designed and conducted experiments with various prompts on the CDR dataset. The experimental results are shown in Fig. 6.

The standard prompts and the prompts from our method are shown in Figs 3 and 4. Specifically, we referred to the work of Kojima *et al.* (2022), which introduced a simple prompt “Let’s think step by step.” for zero-shot tasks, forming the Zero-shot-CoT.

Compared to standard prompts and zero-shot prompts, our method shows more balanced performance across all metrics, with a particularly significant improvement in the *F*1 score. This result further validates the effectiveness of our proposed CoT strategy in enhancing the performance of relation extraction tasks.

**Figure 7.**
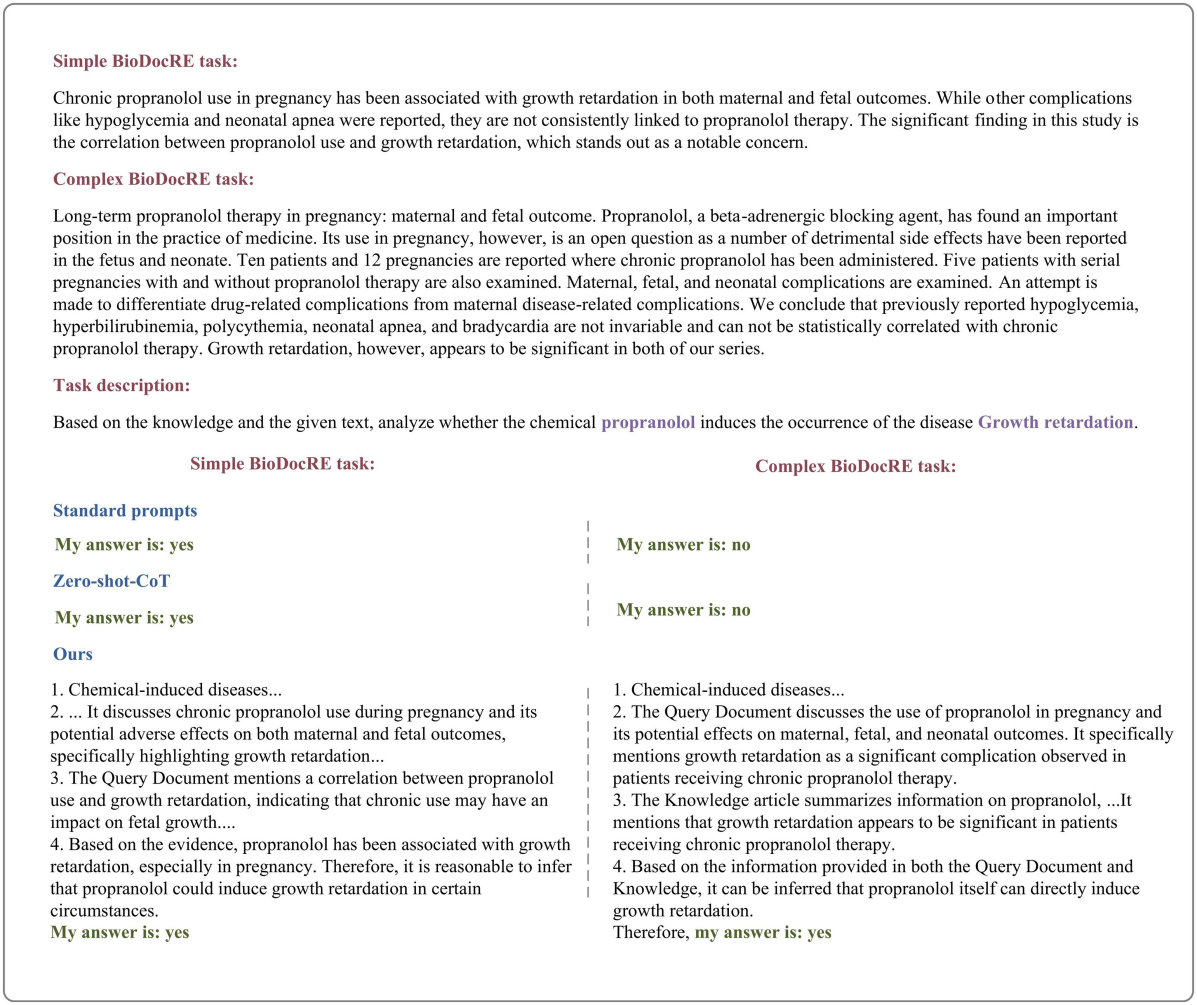
Comparison of different prompts on tasks of varying complexity.

Specifically, compared to the Standard Prompt and Zero-Shot-CoT, our method achieved an improvement of 11.04% and 9.52% in *F*1 score, respectively. Although the Standard Prompt and Zero-Shot-CoT achieved better recall, covering more correct samples, they struggle to handle the complex tasks of BioDocRE. To further illustrate this issue, we tested these three prompts on BioDocRE tasks of varying difficulty, as shown in Fig. 7. The complex BioDocRE task data came from the CDR dataset, while the simple BioDocRE task data were obtained by shortening the text length and highlighting the main content based on the complex BioDocRE task data using GPT-3.5. It can be seen that our prompts are better adapted to various BioDocRE tasks.

### 5.4 Impact of different chunks

In this experiment, we explored the impact of different types and quantities of chunks on the experimental results of the GDA dataset, using a low similarity chunk, zero high similarity chunks, one high similarity chunk, and three high similarity chunks. The experimental results are shown in Table 6.

**Table 6.** Impact of different chunks.

Chunk	*P* (%)	*R* (%)	*F*1(%)
1 low similar chunk	43.88	57.75	49.87
0 chunk	37.78	**73.57**	49.92
1 chunk	**47.08**	66.54	**55.11**
3 chunks	45.30	67.47	54.21

Bold values indicate the best performance in each column.

The results show that using low similarity chunks leads to a decline in model performance, with a 0.05% decrease in *F*1 compared to using zero high similarity chunks. When high similarity chunks are used, the configuration with one chunk performs the best, achieving an *F*1 score of 55.11%. This further indicates that one high similarity chunk allows the SyRACT framework to better adapt to the DocRE task, yielding improved performance. However, when three chunks are used, the performance does not significantly improve, suggesting that an excess of chunks introduces data redundancy.

### 5.5 Case study

**Figure 8.**
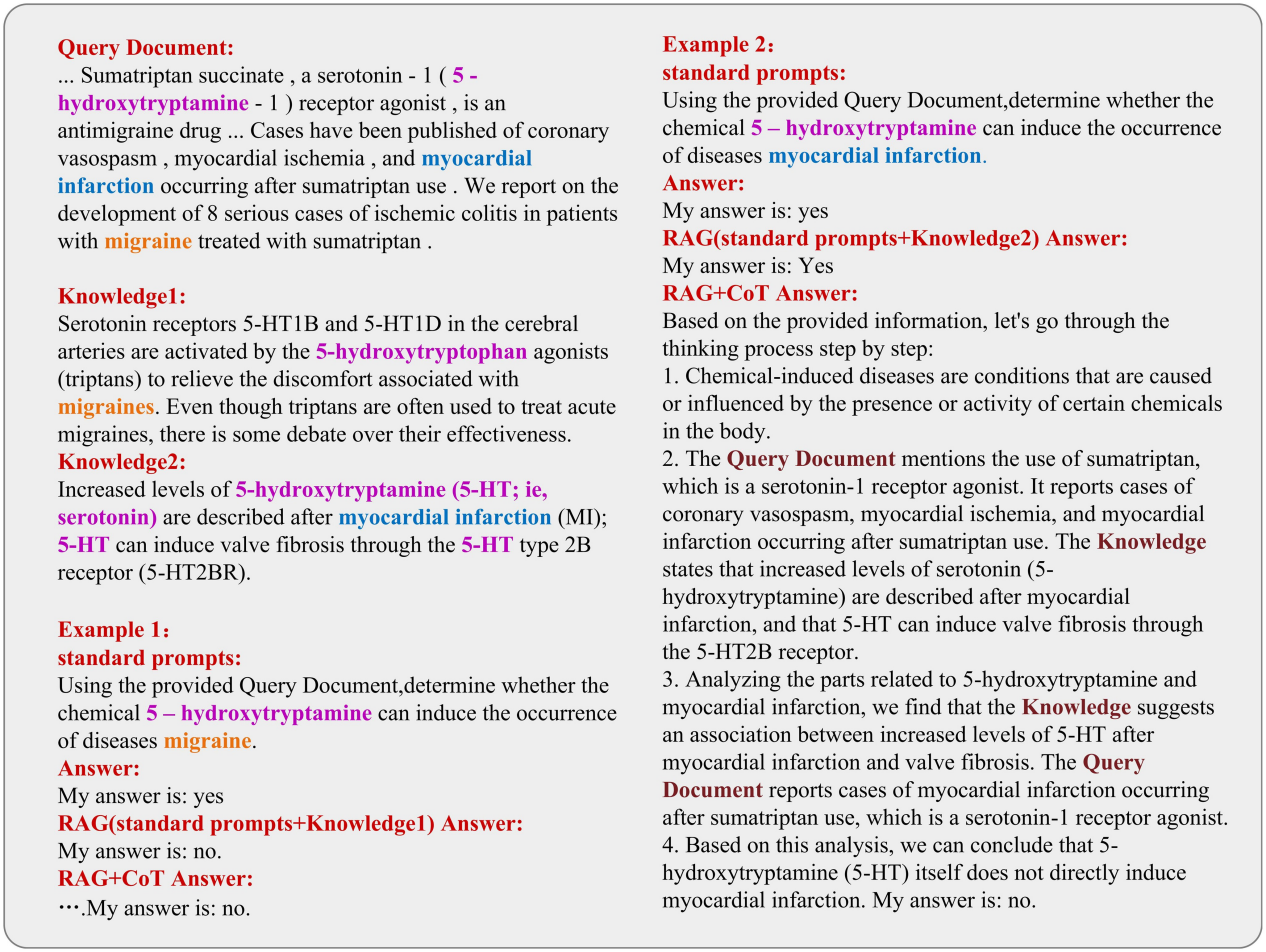
Demonstration of the effectiveness of combining RAG and CoT to improve BioDocRE.

To more intuitively demonstrate the effectiveness of our method, we randomly selected two examples from the CDR dataset and present them in Fig. 8. In Example 1, the model was unable to correctly infer the relation between the compound and the disease when using only standard prompts. However, after incorporating the most relevant text chunk retrieved through RAG (referred to as Knowledge1), the model accurately identified the relation between the entities. This result effectively demonstrates the critical role of RAG in this task.

In contrast to Example 1, in Example 2, the model failed to accurately infer the relation between the entities regardless of whether only standard prompts or additional knowledge (Knowledge2) were used. This indicates that the difficulty of relation extraction between different entities within the same document can vary. However, after integrating CoT, the model successfully extracted the correct relation between the entities. This further underscores the crucial role of CoT in enhancing the model’s reasoning capabilities. Additionally, from the model’s Answer output, we can clearly observe that CoT significantly improved the interpretability of the results, allowing us to better understand the model’s reasoning process.

## 6 Conclusion

We proposes a zero-shot BioDocRE framework that synergizes RAG and CoT, named SyRACT. The framework constructs an external database to introduce relevant knowledge to LLMs, mitigating the hallucination issues that LLMs are prone to, and ensuring the accuracy of the generated content. Meanwhile, the incorporation of the CoT approach significantly enhances the reasoning capabilities of LLMs and improves the interpretability of their outputs, making the process of identifying complex relation more comprehensible. In future research, we plan to enhance the SyRACT framework with more flexible and efficient external knowledge. Additionally, we will explore the potential of SyRACT in tasks such as biomedical named entity recognition and biomedical question answering.

## Data Availability

The source code and dataset of SyRACT have been uploaded to https://github.com/donggggxin/SyRACT.
